# Assessing hearing health inequalities using routine health information systems

**DOI:** 10.1057/s41271-025-00584-8

**Published:** 2025-07-11

**Authors:** Dialechti Tsimpida, Roberta Piroddi, Konstantinos Daras, Gabriella Melis

**Affiliations:** 1 Centre for Research On Ageing, University of Southampton, Murray Building (58), Highfield Campus, Southampton, SO17 1BJ UK; 2 Department of Gerontology, University of Southampton, Murray Building (58), Highfield Campus, Southampton, SO17 1BJ UK; 3 Department of Health Data Science, University of Liverpool, Liverpool, UK; 4 Department of Public Health, Policy and Systems, University of Liverpool, Liverpool, UK; 5 NIHR ARC NWC), National Institute for Health Research Applied Research Collaboration North West Coast, Liverpool, UK; 6 National Disease Registration Service (NDRS), NHS England, UK

**Keywords:** Hearing health, Public health, Inequalities, Spatial statistics, Health policy

## Abstract

Hearing loss is a significant public health challenge, with prevalence estimates based on projected age demographics rather than actual public health needs. This study aimed to quantify hearing loss using real-world data from primary care and explore local patterns and trends from 2013 to 2022 in Cheshire and Merseyside counties of Northwest England. Annual hearing loss prevalence was measured using an ecological space–time analysis of 2.7 million primary care records from Cheshire and Merseyside Integrated Care System. We applied cluster and outlier analysis with geographically weighted regression to examine local deprivation effects. We detected spatial clusters of high prevalence of hearing loss in Cheshire and an increasing trend in hearing loss prevalence in Halton. Deprivation accounted for up to 35% of hearing loss variance in 2020. Monitoring spatial patterns of hearing loss is crucial for addressing health inequalities and guiding targeted prevention and intervention strategies.

**Supplementary Information:**

The online version contains supplementary material available at 10.1057/s41271-025-00584-8.

## Key messages

- Current estimates of hearing loss severity in older adults rely on outdated data from 40 years ago and projected age demographics, highlighting a pressing need for validation.
- This study shows significant inequalities in hearing health in Cheshire and Merseyside counties of North England that persist irrespective of age.
- The findings underscore the importance of measuring and monitoring hearing loss at the population level, advocating the integration of hearing care into national health strategies and policies.

## Introduction

Hearing loss is one of the most pressing public health challenges today, with significant social and economic implications [1, 2]. In England, over 9 million adults are affected, contributing to an annual economic cost exceeding £25 billion annually due to reduced productivity and unemployment [3]. Hearing loss profoundly impacts the quality of life, mental health, work, education, family life, communication, and social engagement [4]. It also increases health and social care costs by its association with multiple chronic health conditions [5].

Current estimates of hearing loss prevalence in England rely on outdated audiological data collected over 40 years ago and projected age demographics [6]. These estimates have not been validated with population-level data, yet continue to guide local hearing health policies [6]. Similar methods of calculating estimated hearing loss prevalence based on the age of the population inform the Global Burden of Disease publications, which then conclude that the prevalence of hearing loss is mainly driven by population growth and ageing [7], creating a circular argument.

Recent compelling evidence has brought to light substantial variations in the prevalence of hearing loss among adults attributed to social and lifestyle factors [8], as well as the geographical location of residence [6]. This revelation underscores the epidemiological inaccuracy of the existing approach for calculating estimates of hearing loss prevalence solely based on age demographics [6]. Recent analyses of the global disease burden of hearing loss are still based on the age-prevalence model rather than updated records [7], which may dramatically underestimate the magnitude of the prevalence and incidence of hearing loss. Notably, recent analyses using the nationally representative National Study of Hearing in England revealed that nearly 200,000 more people aged 50 and above in England are estimated to have hearing loss compared to current estimates in the NHS hearing loss data tool [6].

While policymakers in England diligently analyse primary care records of individuals with recorded vision loss and monitor population-level changes annually, similar attention to hearing loss data is lacking [9], even though hearing loss has been recognised as the largest potentially modifiable risk factor for dementia, is strongly associated with cognitive decline, and leads to poor mental health [10, 11].

This study addresses these gaps using primary care records to quantify the recorded hearing loss (as the partial or total inability to hear sounds) in older adults and explore patterns and trends independent of age demographics.

## Data and methods

In this study, we focussed on the Cheshire and Merseyside Integrated Care System (ICS) in England, which consists of 1,562 Lower Super Output Areas (LSOAs) with an average population of 1500 people [12]. We used the LSOA boundaries published by the Office for National Statistics as of 21 March 2021 [12] and the digital vector boundaries for Integrated Care Boards, in England, as at April 2023 to compare sub-integrated care board locations in Cheshire and Merseyside ICS(Cheshire, Halton, Knowsley, Liverpool, South Sefton, Southport and Formby, St Helens, Warrington, Wirral).

We utilised the Combined Intelligence for Population Health Action (CIPHA) shared dataset, an individual-level, linked dataset established during the COVID-19 pandemic for continuously updated population health management [13]. Covering 97% of the registered population (~ 2.7 million people), this dataset includes demographic characteristics, primary care records, and LSOA-based residential information updated as patients notify general practitioners (GPs) of address changes. To estimate population sizes, we linked this dataset to the Office for National Statistics mortality register.

We then linked primary care records of all GP consultations from the beginning of year 2013 to the end of 2022 to estimate the prevalence and incidence of hearing loss over the past decade. We calculated hearing loss prevalence using Systematized Nomenclature of Medicine Clinical Terms (SNOMED) codes capturing all hearing loss types, from 2013 to 2022. For this study, we combined all available SNOMED codes on hearing loss to generate time series estimates of hearing loss prevalence for each LSOA over the study period.

A disclosure risk assessment was conducted following the ISB1523 Anonymisation Standard for Publishing Health and Social Care Data. To protect privacy, any numerators below five were suppressed if the denominator was ≤ 1,000 cases. The full list of codes and descriptions, including suppressed hearing loss types, is provided in Supplementary Material Table S1.

To account for differences in age structure across populations, we applied a rigorous age-adjustment process. The population was stratified into specific age groups (51–60, 61–70, 71–80, and 81 +), and hearing loss rates were calculated for each age group within each LSOA. These rates were then standardised using the age distribution of a standard population to derive a weighted average rate per LSOA and year. This adjustment was crucial to ensure that observed variations in hearing loss rates reflected true differences in prevalence rather than differences in the underlying population age composition.

Using age-adjusted prevalence estimates, we calculated the annual aggregate hearing loss diagnoses per LSOA by taking the weighted average count of the number of patients aged 50 years and older diagnosed with any type of hearing loss per LSOA and dividing it by the mid-year population estimates of individuals aged 50 and older within each LSOA. This approach ensured that the final rates accounted for both age structure differences and population size variations across areas and over time, enabling meaningful comparisons of hearing loss prevalence.

To provide additional context and test the spatial association with hearing loss prevalence, we incorporated the Index of Multiple Deprivation (IMD), a widely used measure of relative deprivation in the United Kingdom. We used the most recent English Index of Multiple Deprivation (IMD 2019), which provides detailed measures of deprivation across England [14] at high spatial resolution, or in small areas.

### Analytical approach

In a univariate analysis, the prevalence of hearing loss was described for each year using central tendency measures (mean and median) and dispersion measures (range, standard deviation, variance, minimum and maximum values). To assess spatial autocorrelation [15] across the region, we applied the Global Moran’s I statistic [16] for each year to measure spatial autocorrelation in values of hearing loss and to test whether the observed pattern was clustered, dispersed, or random.

Guided by the results of Global Moran’s I, we performed Cluster and Outlier Analysis, using the Anselin Local Moran’s I algorithm to identify local indicators of spatial association (LISA) and correct for spatial dependence [17]. The LISA identified statistically significant spatial clusters of small areas with high values (high/high clusters) and low values (low/low clusters) of hearing loss, as well as high and low spatial outliers—where a high value is surrounded by low values (high/low clusters) and vice versa (low/high clusters). Extending our spatial methods, we conducted a spatiotemporal analysis [18] of total hearing loss prevalence per LSOA for which there were no suppressed values; this analysis used an ecological space–time implementation of the Anselin Local Moran’s I statistic to identify statistically significant clusters and outliers in the context of both space and time [18].

We then created a set of three clusters based on the similarity of time series values. These clusters reflected approximately equal values across time, representing high, medium, and low rates of increase. Selecting three clusters was deemed optimal for supporting ongoing research examining the link between hearing loss and the three clusters of high, medium, and low rates of depression, as described in our recently published work [19].

To predict hearing loss trends from 2023 to 2027, we applied Curve Fit 5-year Forecast, assuming a continuation of trends observed from 2013 to 2022. Multiple forecasted space–time cubes were compared and merged to identify the best forecast for each location based on the Validation Root Mean Square Error (RMSE), assessing linear, parabolic, S-shaped (Gompertz), and exponential curve types. For the validation process, we used the Auto-Detect option for the Curve Type parameter, which fit all four curve types at each location and selected the one with the smallest Validation RMSE.

To understand the local effects of deprivation (IMD 2019) on hearing loss in 2020, we applied the Geographically Weighted Regression (GWR) model, which is suitable for spatial distributions exhibiting statistically significant non-stationarity [18, 20]. GWR is a local regression model that constructs a single equation for each feature in the study area using only its neighbouring features, allowing variable relationships to change across space. As a result, GWR produced the local R-squared values for each feature in the study area.

Statistical significance was set at the 99% confidence level to enhance the robustness and stringency of our results. Analyses were performed in ArcGIS Pro Version 2.9.2 [21] using the following tools, in order of execution: Spatial Join tool, Spatial Autocorrelation tool, Optimised Outlier Analysis tool (999 permutations), Space–Time Cube Creation tool, Space–Time Pattern Analysis tool, Evaluate Forecasts By Location tool, Time Series Clustering tool, and Tabulate Intersection tool.

## Results

Table 1 shows the prevalence of hearing loss in Cheshire and Merseyside ICS region from 2013 to 2022. During the study period, 219,068 patients were recorded with hearing loss issues. A detailed breakdown of records for each SNOMED code is provided in Table S1 of the supplemental material. The absolute range of hearing loss prevalence among LSOAs in the region increased from 9.54 to 15.20 percent from 2013 to 2022, representing a 59.3% rise in hearing loss over the past decade. Also, the variance increased from 1.36 to 5.12 over the study period, indicating a rise in the spread of prevalence scores from the mean value in each consequent year, showing widening hearing health inequalities.

**Table 1:** Summary Statistics of Hearing Loss Prevalence in Cheshire and Merseyside Integrated Care System (ICS): 2013–2022

Year	Min	Max	Mean	Standard deviation	Median	Range	Variance
2013	0.49	10.03	3.31	1.17	3.19	9.54	1.36
2014	0.65	10.74	3.76	1.28	3.64	10.09	1.63
2015	0.65	11.24	4.24	1.39	4.13	10.59	1.93
2016	0.72	12.33	4.72	1.52	4.60	11.61	2.31
2017	0.78	13.30	5.19	1.64	5.10	12.52	2.69
2018	0.84	13.75	5.66	1.75	5.62	12.91	3.08
2019	0.97	14.53	6.12	1.87	6.03	13.56	3.51
2020	1.07	15.15	6.63	2.02	6.57	14.08	4.07
2021	1.12	15.47	6.98	2.13	6.89	14.35	4.54
2022	1.16	16.36	7.39	2.26	7.26	15.20	5.12

The results of the Global Moran’s I statistic, shown in Table S2, indicate strong clustering of hearing loss prevalence across all years from 2013 to 2022. This finding demonstrated that the observed values of hearing loss were not randomly distributed in the LSOAs but exhibited a significant spatial correlation. The Global Moran’s I correlation coefficient, measuring the similarity of one value to those surrounding it, ranged from 0.55 to 0.59 during this period. This clustering pattern had a less than 1% likelihood of occurring by random chance, as indicated by the z-scores [23].

**Fig. 1:**
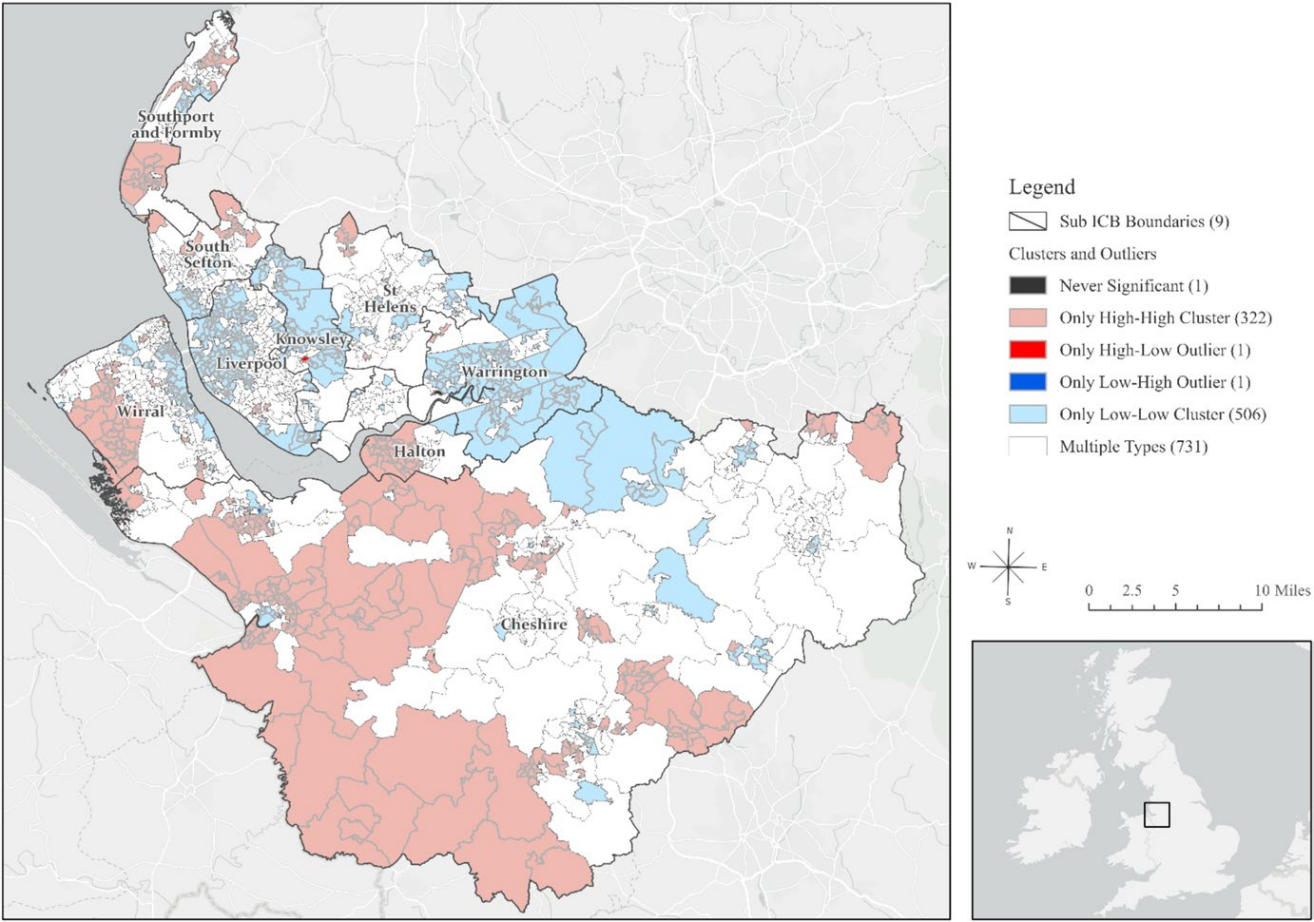
Space–Time Analysis: Anselin Local Moran's I Statistic for the Prevalence of Hearing Loss in Adults Aged 50 and Above in Cheshire and Merseyside Integrated Care System (ICS) (2013–2022) [24]

The LISA analysis revealed statistically significant spatial clusters, including high values clusters (high/high), low values clusters (low/low), and outliers (high/low and low/high). The ecological space–time implementation of the Anselin Local Moran’s I statistic is illustrated in Fig. 1. Details of the area coverage (in m^2^), the estimated population residing, and the percentages of coverage and population per cluster in each region are shown in Table S3 of the supplemental material. Cheshire demonstrated the highest concentration of high-high clusters of hearing loss, with 42% of the area having high values surrounded by high values in both space and time, and an estimated 43% of the population living in those areas (*n =* 138,298 people). In contrast, Liverpool was the sub-integrated care board location with the lowest percentage of the area (0.95%) and population (1.7%, *n =* 2,647) living in high-high clusters.

**Fig. 2:**
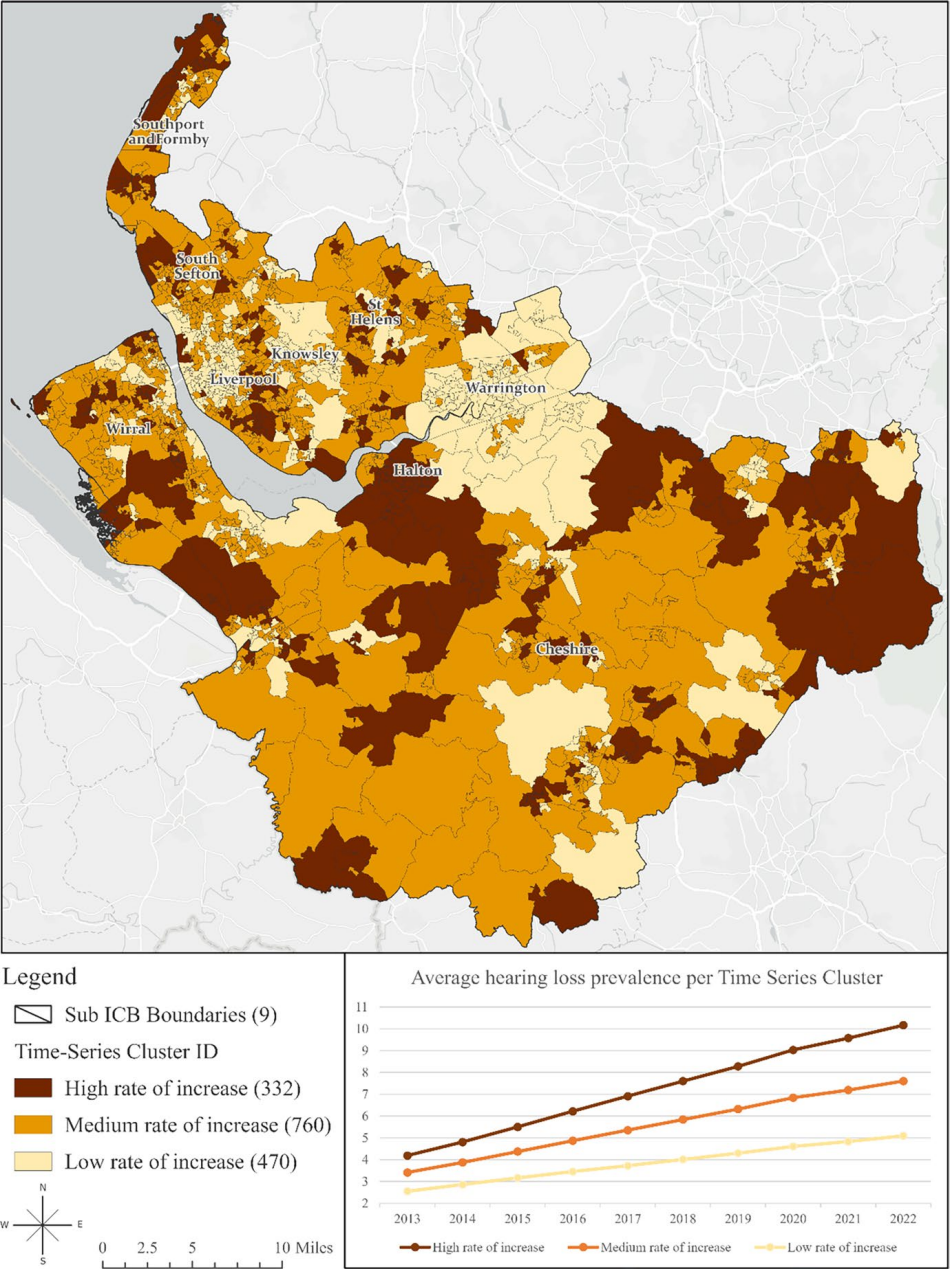
Time Series Clustering Analysis: Exploring the Patterns of Hearing Loss in Adults (50 +) in Cheshire and Merseyside Integrated Care System (ICS), 2013–2022 [25]

Figure 2 presents the results of the Time Series Clustering tool, which identified clusters based on the similarity in the trends of time series values of the prevalence of hearing loss derived from the Anselin Local Moran’s I algorithm. Across all clusters—whether high, medium, or low rates of increase—the prevalence of hearing loss has shown consistent growth since 2013. Hearing health inequalities are increasingly widening over time, particularly in certain regions where populations experience a more rapid decline in hearing health at an accelerated rate. The area coverage (in m^2^), the estimated population residing, and the percentages of coverage and population per cluster associated with labels ‘low,’ ‘medium,’ and ‘high rate of increase’ in each region are provided in Table S4 of the supplemental material. The Halton region had the highest percentage of areas with a high rate of increase (40.38%), with nearly half of its population (54.29%, *n =* 27,369) residing in these areas. In comparison, only 1.72% of the population in Warrington (*n =* 1,440) lived in areas with a high rate of increase.

**Fig. 3:**
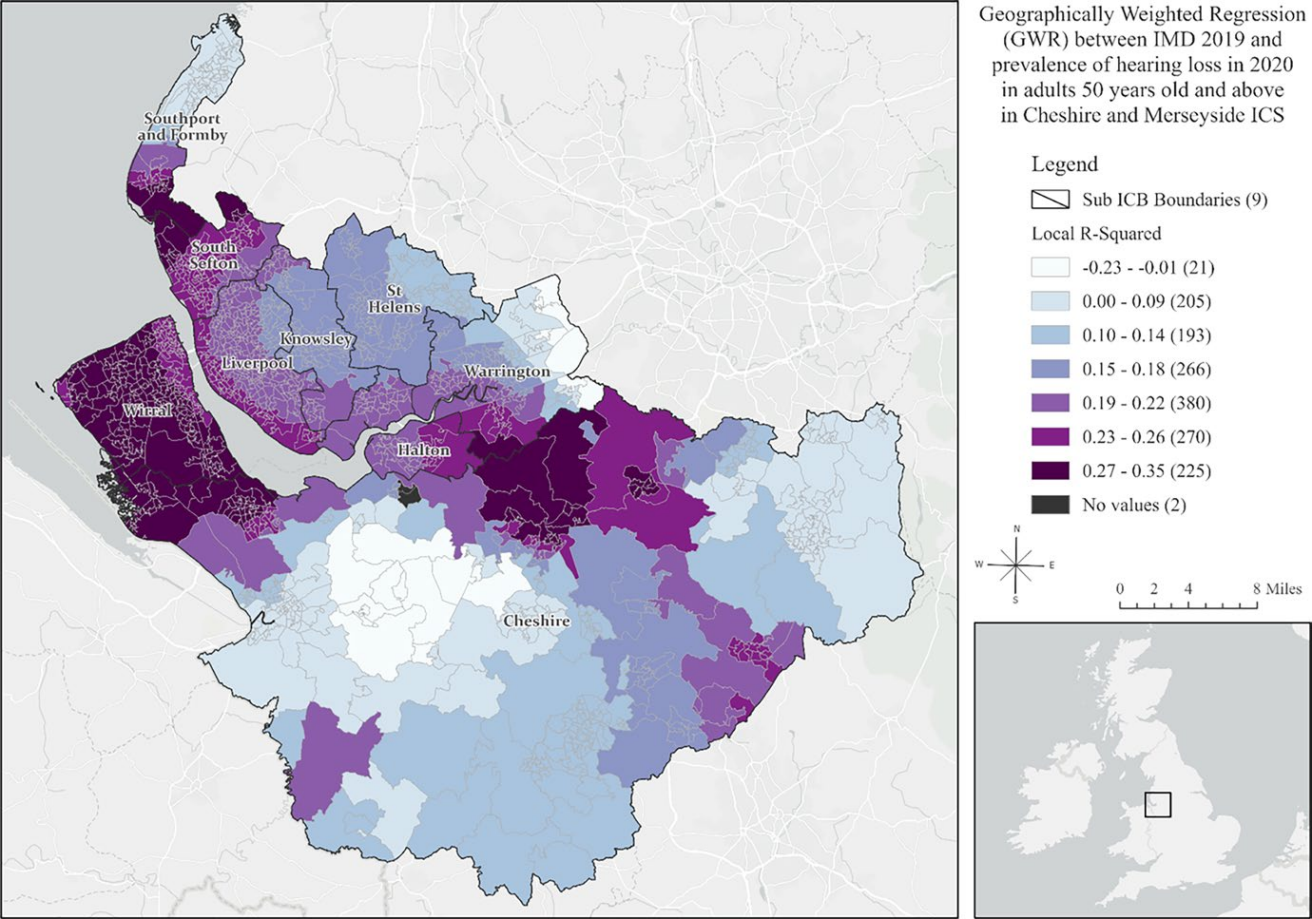
Geographically Weighted Regression Analysis: Examining the Relationship between Index of Multiple Deprivation (IMD) 2019 and Prevalence of Hearing Loss in Adults (50 +) in Cheshire and Merseyside ICS, 2020 [28]

The Koenker (BP) Statistic indicated statistically significant non-stationarity, suggesting that the relationship between IMD and hearing loss varies across the ICS. Fig. S1 [26] and Fig. S2 [27] in the supplemental material provide a bivariate display of IMD 2019 and the prevalence of hearing loss in adults aged 50 years old and above in Cheshire and Merseyside ICS in 2020. The results of GWR between median age in each LSOA and hearing loss prevalence in adults 50 + in 2020 are also presented. Figure 3 shows the results of the GWR analysis between the Index of Multiple Deprivation (IMD) 2019 and hearing loss prevalence in adults 50 years old and above in Cheshire and Merseyside ICS in 2020. The darker areas do not indicate where the highest deprivation or highest hearing loss prevalence; rather, they highlight where the relationship between IMD and hearing loss was strongest, as identified through GWR [18].

**Fig. 4:**
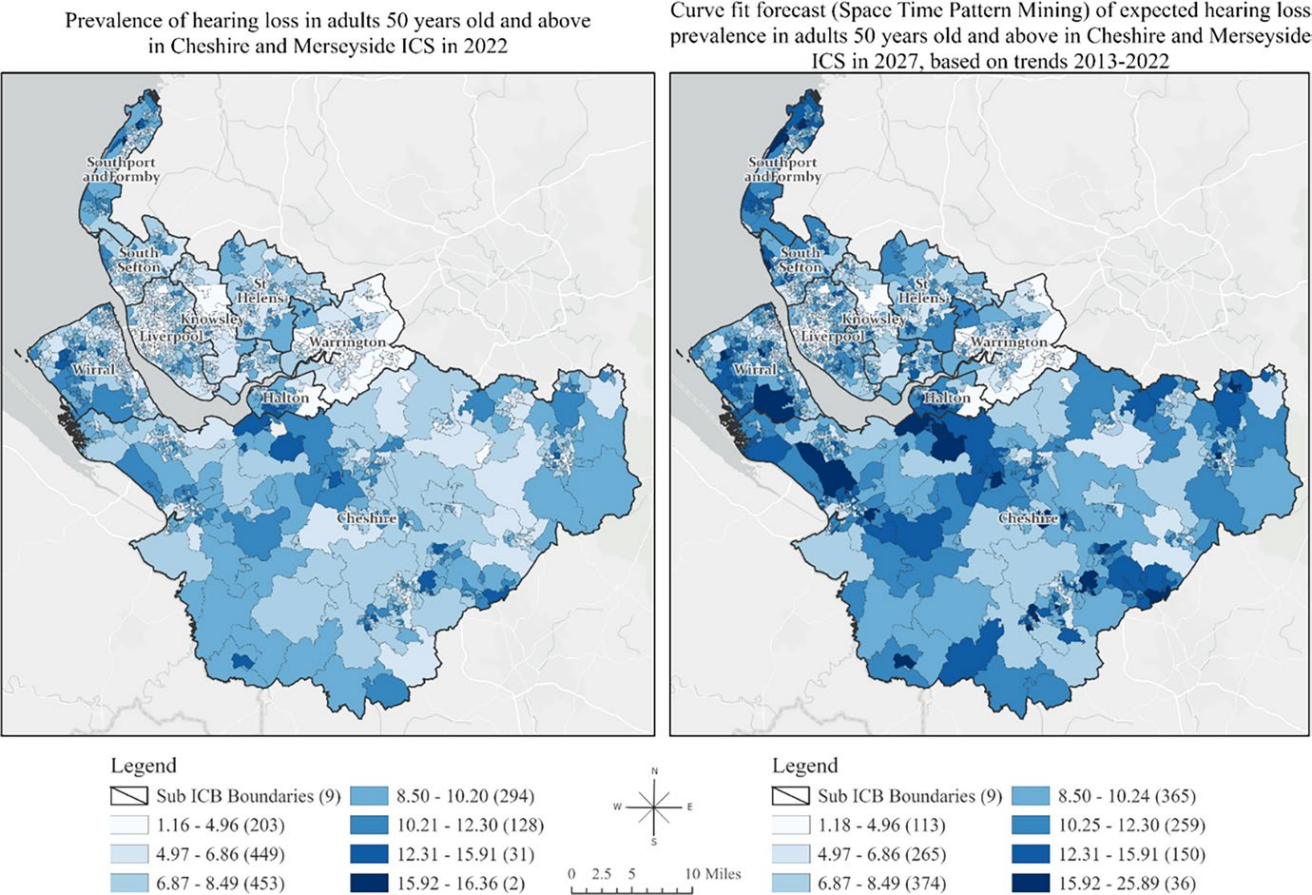
Prevalence of Hearing Loss in Adults (50 +) in 2022 and Projected Prevalence of Hearing Loss in 2027: A Curve Fit Forecast Based on Decade-long Trends (2013–2022) [29, 30]

The local R-squared values varied across the ICS, suggesting that IMD is a strong predictor of hearing loss in some areas, explaining up to 35% of the variance in prevalence in 2020. Summarised results of GWR between IMD 2019 and hearing loss prevalence in 2020 for each region are shown in Table S5 of the Supplemental Material. Additionally, Table S6 provides summary statistics of the median age among LSOAs in the nine Sub Integrated Care Board Locations within the ICS 2020, while Table S7 presents the results of GWR analysis between median age and hearing loss prevalence in 2020. Figure 4 shows the prevalence of hearing loss in adults 50 years old and above in 2022, alongside the forecasted prevalence in 2027, based on trends observed over the last decade. Detailed forecasted statistics are shown in Table S8 of the Supplemental Material.

## Discussion

This study utilised continuously updated records from Cheshire and Merseyside CIPHA, linking for the first time individual-level primary care data to population-level quantification of hearing loss prevalence. The findings also revealed stark inequalities in hearing health beyond the ageing effect.

Cheshire had the highest concentration of High-High clusters of hearing loss, meaning that during the past 10 years, this county consistently experienced high prevalence of hearing loss, encompassing an estimated 43% of the population.​ In contrast, Halton experienced the most rapid increase in hearing loss rates, with 40.4% of its areas showing a high rate of increase over time. Nearly half of Halton's population (54.3%, *n =* 27,369) resided in areas with this accelerated decline in hearing health. Importantly, deprivation emerged as a strong predictor of hearing loss, explaining up to 35% of the variance in hearing loss across Cheshire and Merseyside ICS in 2020.

Previous studies often focussed on small, selected samples of individuals with hearing loss from specific primary care settings [31–34] or evaluated hearing data from specific primary care centres engaged in an Older People Health Care Programme [35]. A comprehensive, population-level analysis of hearing loss using primary care records was notably absent.

In England, prevalence estimates for hearing loss that inform the NHS Hearing Loss Data Tool [36] rely on small, outdated datasets. These datasets, derived from only 1,538 individuals in the 1980s [37], lacked national representativeness and provided limited utility for local service planning. Recent survey data highlighted the inaccuracies of existing hearing loss estimates [6]. However, aside from projections and broad estimates, no prior research has quantified the actual severity of hearing loss using updated data, leaving a critical gap that this study addresses [6].

### Strengths and limitations

This study leveraged records from all practices in the Cheshire and Merseyside, covering 2.7 million individuals. By using spatial analysis, we were able to identify clustering patterns, locate local hotspots, and uncover localised risk factors—insights that traditional regression models could not provide. We also opted for the 99% statistical significance level—rather than the more conventional 95%—to enhance the robustness of our results. This higher significance level provided a more stringent criterion for accepting the statistical relationships observed, reducing the likelihood of Type I errors and increasing confidence in the significance of our findings [38].

Nevertheless, the study has limitations. The reliance on GP-recorded diagnoses may introduce bias due to inconsistent documentation or underreporting. In the absence of a nationwide screening programme for early detection of adult hearing loss through routine primary care health checks, it is plausible that instances of hearing loss are likely underreported by patients [39], and their documentation within patient histories by practitioners might lack consistency [40]. There is a possibility that GPs may not identify all patients who could benefit from treatment, which could result in incomplete or inconsistent clinical records [19]. Additionally, GPs' decisions to diagnose hearing loss might be influenced by personal biases or preferences, leading them to document symptoms instead of referring patients for audiological assessment, where a formal diagnosis could be made and recorded on patients' records [39]. Moreover, individuals with the partial hearing loss might not routinely consult GPs, leading to an omission of milder cases from primary care records or their non-recording by GPs [39]. Consequently, the prevalence of recorded hearing loss is prone to underestimating the actual prevalence at the population level [41].

Another limitation of our analysis is the ecological nature of the relationship assessment between deprivation and hearing loss. Although we observed temporal alignment, our analysis lacks individual measures both cross-sectionally and longitudinally. This limitation affects the generalisability of our findings regarding the relationship between hearing loss and IMD across various spatial contexts. We recommend that future research incorporates a longitudinal approach, particularly when the updated Index of Multiple Deprivation (IMD) is released. The anticipated update of the IMD, with a provisional release date of late 2025 [42], emphasises the need for ongoing research to deepen our understanding of how the dynamics between hearing loss and deprivation evolve over time and across different regions.

### Research and policy implications

Reflecting the sentiments of the *World Report on Hearing* [4], our research aligns with the World Health Organization's (WHO) recommendations to address the public health dimensions of hearing loss comprehensively and supports the WHO's call for proactive, evidence-based policies to tackle hearing loss. The emphasis on screening for hearing loss [31] and related conditions remains central to this strategy, underscoring the need for effective and sustainable screening protocols that contribute to functional well-being and enhanced quality of life. Considering the rapid increase in hearing loss in specific locations, additional research is necessary to investigate environmental risk factors, such as noise pollution, that could exacerbate hearing health inequalities in these areas, regardless of people’s age [43]. Future assessments should consider the varying degrees of disability associated with hearing loss to inform targeted policy decisions and evaluate integrated care models in areas with high rates of both hearing loss and depression [19].

The insights gained from this study can significantly inform health policy, especially in regions like the North West and North East, where the hearing loss burden is pronounced [6]. The evidence supports integrating hearing health indicators within the Public Health Outcomes Framework [9], as highlighted in submissions to the UK Parliament [41], underscoring the importance of systematically collecting, analysing, and interpreting available hearing-related data in alignment with other chronic health conditions. Moreover, it reinforces the need to include hearing assessments within NHS Health Check.

Through collaboration with policymakers and co-production of a Policy Brief with Place Directors in the Cheshire and Merseyside Integrated Care System [44], this study influenced policy development. Notably, it led to the inclusion of hearing loss data collection among the recommendations of the Chief Medical Officer’s Annual Report 2023: Health in an Ageing Society [45]. The report highlighted a critical gap: epidemiological data on health conditions, such as hearing loss and mental health, that contribute to disability in older adults are not routinely collected. To enable effective planning, it is essential for organisations such as the NHS, Office for National Statistics (ONS), and both central and local government to systematically collect and share data on the health and care needs of older adults, disaggregated by factors such as ethnicity, sex, and other protected characteristics.

Furthermore, the implications of our research extend beyond national boundaries, emphasising the global significance of quantifying and monitoring hearing loss at a population scale, using individual-level data [4]. Consequently, quantifying and monitoring hearing loss prevalence on a population scale underscores the imperative of integrating hearing care within national health agendas. Moving forward, the estimation of the global burden of disease related to hearing loss should be based on national-level primary care records. This is particularly important given the regional disparities in hearing loss prevalence among adults of similar age [46].

## Conclusions

This study leveraging primary care records in Cheshire and Merseyside ICS in England establishes a novel approach for quantifying hearing loss prevalence at the population level with individual-level data. Deprivation emerged as an important factor in hearing loss variation. These findings emphasise the importance of integrating hearing care into local and national health strategies and policies. Future research should investigate the factors driving higher prevalence of hearing loss, including environmental, behavioural, and sociodemographic factors, and develop tailored public health interventions.

## Supplementary Information

Below is the link to the electronic supplementary material.

Supplementary file1 (DOCX 1684 KB)

## Data Availability

The authors confirm that the data supporting the findings of this study are available within the article and its supplementary materials.
