# Supplementary material for: Assessing hearing health inequalities using routine health information systems
Source: J Public Health Policy. 2025 Jul 11;46(3):630–44. doi: 10.1057/s41271-025-00584-8 (PMC12328234; doi:10.1057/s41271-025-00584-8)
Supplement: Supplementary file 1 — Supplementary file1 (DOCX 1684 KB) [file 41271_2025_584_MOESM1_ESM.docx]

Original Article

Assessing Hearing Health Inequalities using Routine Health Information Systems

**Dialechti Tsimpida ^1,2,^*, Roberta Piroddi ^3^, Konstantinos Daras ^4,5,^*, Gabriella Melis ^6^**

^1^ Centre for Research on Ageing, University of Southampton, UK; [d.tsimpida@soton.ac.uk](mailto:d.tsimpida@soton.ac.uk)

^2^ Department of Gerontology, University of Southampton, UK; [d.tsimpida@soton.ac.uk](mailto:d.tsimpida@soton.ac.uk)

^3^ Department of Health Data Science, University of Liverpool, UK; [r.piroddi@liverpool.ac.uk](mailto:r.piroddi@liverpool.ac.uk)

^4^ Department of Public Health, Policy and Systems, University of Liverpool, UK

^5^  National Institute for Health Research Applied Research Collaboration North West Coast (NIHR ARC NWC), UK; [konstantinos.daras@liverpool.ac.uk](mailto:konstantinos.daras@liverpool.ac.uk)

^6^  National Disease Registration Service (NDRS), NHS England, UK; [gabriella.melis1@nhs.net](mailto:gabriella.melis1@nhs.net)

***** Correspondence: [d.tsimpida@soton.ac.uk](mailto:d.tsimpida@soton.ac.uk)

**Supplemental Material**

Table of contents

**Table S1.** Systematized Nomenclature of Medicine Clinical Terms (**SNOMED) Codes for Recording Various Types of Hearing Loss** ..........................................................................................………………………..(page 3)

**Table S2. Global Moran's I Statistic: Hearing Loss Prevalence in Cheshire and Merseyside ICS, 2013-2022.**……………………………………………………………….……………………………………… (page 5)

**Table S3. Space-Time Pattern Mining of Hearing Loss Prevalence: Area Coverage and Estimated Population in Cheshire and Merseyside ICS Based on Anselin Local Moran's I Statistic of Lower Super Output Areas (LSOAs), 2013-2022**……………….…………………………………………………….. (page 6)

**Table S4. Temporal Analysis of Hearing Loss Prevalence: Area Coverage and Estimated Population in Cheshire and Merseyside ICS Based on Time Series Clustering of Lower Super Output Areas (LSOAs), 2013-2022**…………………………………………………………………………...…………………….. (page 7)

**Table S5. Geographically Weighted Regression Analysis: Exploring the Relationship between Index of Multiple Deprivation (IMD) 2019 and Prevalence of Hearing Loss in 2020**………………………….. (page 8)

**Table S6. Summary Statistics of Median Age in Lower Super Output Areas (LSOAs): Cheshire and Merseyside ICS Sub Integrated Care Board Locations, 2020**……………...……..………………….. (page 8)

**Table S7. Geographically Weighted Regression Analysis: Investigating the Relationship between Median Age of Lower Super Output Areas (LSOAs) in Cheshire and Merseyside ICS Sub Integrated Care Board Locations and Prevalence of Hearing Loss in 2020**………………………………...…………………..(page 8)

**Table S8. Summary Statistics of Hearing Loss Prevalence in Sub Integrated Care Board Locations within Cheshire and Merseyside ICS: 2013-2027**………………………………..……………..………...…….(page 9)

**Fig. S1. Bivariate Analysis: Mapping IMD 2019 and Prevalence of Hearing Loss in Adults (50+) in Cheshire and Merseyside ICS, 2020**…………………………………………..……..………...………………… (page 18)

**Fig. S2. Geographically Weighted Regression Analysis: Exploring the Relationship between Median Age in LSOAs and Hearing Loss Prevalence in Adults (50+) in Cheshire and Merseyside ICS, 2020**…………………………………………………………………………..………...…………..……..(page 19)

**Table S1.** Systematized Nomenclature of Medicine Clinical Terms (**SNOMED) Codes for Recording Various Types of Hearing Loss**

| **N** | **Code (SNOMED)** | **Description** |
| --- | --- | --- |
| 8359 | 272033007.00 | deafness symptom |
| 971 | 737047001.00 | mild acquired hearing loss |
| 176 | 194429000.00 | mix conductive and sensorineural hearing loss bilateral |
| 127 | 73371001.00 | neural hearing loss |
| 1343 | 77507001.00 | mixed conductive and sensorineural hearing loss |
| 136 | 700454004.00 | profound sensorineural hearing loss |
| 39 | 61947007.00 | deaf mutism |
| 54 | 204224006.00 | ear anomalies with hearing impairment |
| 39 | 194415001.00 | conductive hearing loss due to a disorder of the middle ear |
| * | 194414002.00 | conductive hearing loss due to a disorder of the tympanic membrane |
| 150 | 42538001.00 | low-frequency deafness |
| 2036 | 343087000.00 | partial deafness |
| * | 194416000.00 | conductive hearing loss due to disorder of inner ear |
| 588 | 860851000000105.00 | mild sensorineural hearing loss |
| 85 | 877191000000104.00 | bilateral profound sensorineural hearing loss |
| 81 | 232325008.00 | chronic deafness |
| 14199 | 285055002.00 | uses hearing aid |
| 132 | 877211000000100.00 | bilateral congenital sensorineural hearing loss |
| 995 | 194417009.00 | conductive hearing loss bilateral |
| 122 | 737050003.00 | profound acquired hearing loss |
| 11 | 267677007.00 | ototoxicity deafness |
| * | 68467004.00 | central hearing loss |
| 293 | 85571008.00 | sensory hearing loss |
| * | 275482009.00 | drug ototoxicity - deafness |
| 9198 | 194424005.00 | sensorineural hearing loss bilateral |
| 262 | 860801000000109.00 | moderate sensorineural hearing loss |
| 104 | 860811000000106.00 | severe sensorineural hearing loss |
| 44 | 95828007.00 | congenital deafness |
| * | 194418004.00 | conductive hearing loss, unilateral |
| * | 194413008.00 | conductive hearing loss due to disorder of the external ear |
| 9275 | 44057004.00 | conductive hearing loss |
| 14393 | 60700002.00 | sensorineural hearing loss |
| * | 194419007.00 | combined conductive hearing loss |
| 149002 | 15188001.00 | hearing loss |
| 308 | 73415002.00 | noise-induced hearing loss |
| * | 194428008.00 | mixed conductive and sensorineural hearing loss |
| 25 | 194425006.00 | sensorineural hearing loss, unilateral |
| 48 | 275259005.00 | congenital malformation of the ear |
| 248 | 737048006.00 | moderate acquired hearing loss |
| 39 | 737049003.00 | severe acquired hearing loss |
| * | 699238006.00 | congenital prelingual deafness |
| 2985 | 118230007.00 | hearing finding |
| * | 186570004.00 | rubella deafness |
| 2331 | 162344009.00 | bilateral deafness |
| 824 | 232326009.00 | high-frequency deafness |

* N<10 was indicated with an asterisc for anonumisation purposes.

**Table S2. Global Moran's I Statistic: Hearing Loss Prevalence in Cheshire and Merseyside ICS, 2013-2022**

| **Year** | **Index** | **Z-score** | **P-value** |
| --- | --- | --- | --- |
| 2013 | 0.598 | 39.057 | 0.000 |
| 2014 | 0.590 | 38.586 | 0.000 |
| 2015 | 0.581 | 37.990 | 0.000 |
| 2016 | 0.568 | 37.179 | 0.000 |
| 2017 | 0.565 | 36.952 | 0.000 |
| 2018 | 0.571 | 37.350 | 0.000 |
| 2019 | 0.574 | 37.543 | 0.000 |
| 2020 | 0.581 | 37.983 | 0.000 |
| 2021 | 0.568 | 37.162 | 0.000 |
| 2022 | 0.558 | 36.529 | 0.000 |

**Table S3. Space-Time Pattern Mining of Hearing Loss Prevalence: Area Coverage and Estimated Population in Cheshire and Merseyside ICS Based on Anselin Local Moran's I Statistic of Lower Super Output Areas (LSOAs), 2013-2022.**

| **Sub Integrated Care Board Locations^1^** | **Category** | **Area km^2^** | **Percentage of area** | **Estimated population of 50 years old and over residing in those areas^2^** | **Percentage of the population 50 years old and over residing in those areas** |
| --- | --- | --- | --- | --- | --- |
| Cheshire | Multiple Types | 1047.504787 | 50.21 | 146826 | 45.6% |
|  | Only High-High Cluster | 876.210121 | 42.00 | 138298 | 43.0% |
|  | Only Low-High Outlier | 0.22107 | 0.01 | 405 | 0.1% |
|  | Only Low-Low Cluster | 159.020002 | 7.62 | 36246 | 11.3% |
| Halton | Multiple Types | 53.340476 | 67.44 | 29699 | 58.9% |
|  | Only High-High Cluster | 21.226873 | 26.84 | 18116 | 35.9% |
|  | Only Low-Low Cluster | 4.527463 | 5.72 | 2594 | 5.1% |
| Knowsley | Multiple Types | 29.757721 | 34.41 | 17970 | 30.9% |
|  | Only High-Low Outlier | 0.356717 | 0.41 | 773 | 1.3% |
|  | Only Low-Low Cluster | 56.354972 | 65.17 | 39360 | 67.7% |
| Liverpool | Multiple Types | 56.108134 | 50.17 | 79981 | 50.5% |
|  | Only High-High Cluster | 1.063003 | 0.95 | 2647 | 1.7% |
|  | Only Low-Low Cluster | 54.672978 | 48.88 | 75728 | 47.8% |
| South Sefton | Multiple Types | 50.474472 | 66.78 | 45261 | 66.2% |
|  | Only High-High Cluster | 15.24368 | 20.17 | 11678 | 17.1% |
|  | Only Low-Low Cluster | 9.24926 | 12.24 | 11425 | 16.7% |
| Southport and Formby | Multiple Types | 45.892548 | 56.66 | 26230 | 46.3% |
|  | Only High-High Cluster | 28.91785 | 35.70 | 23866 | 42.2% |
|  | Only Low-Low Cluster | 4.430794 | 5.47 | 6515 | 11.5% |
| St Helens | Multiple Types | 110.159203 | 80.77 | 54493 | 72.7% |
|  | Only High-High Cluster | 5.53329 | 4.06 | 3465 | 4.6% |
|  | Only Low-Low Cluster | 20.688873 | 15.17 | 16959 | 22.6% |
| Warrington | Multiple Types | 42.012023 | 23.26 | 14955 | 17.8% |
|  | Never Significant | 0.380614 | 0.21 | 661 | 0.8% |
|  | Only High-High Cluster | 0.979677 | 0.54 | 1194 | 1.4% |
|  | Only Low-Low Cluster | 137.250772 | 75.99 | 67082 | 80.0% |
| Wirral | Multiple Types | 91.730879 | 57.00 | 77846 | 55.8% |
|  | Only High-High Cluster | 44.770117 | 27.82 | 34341 | 24.6% |
|  | Only Low-Low Cluster | 20.413506 | 12.69 | 27204 | 19.5% |

^1^ Sub Integrated Care Board Location definition: <https://www.datadictionary.nhs.uk/supporting_information/sub_integrated_care_board_location.html#:~:text=A%20Sub%20Integrated%20Care%20Board%20Location%20is%20a,purposes%20and%20to%20support%20the%20ELECTRONIC%20HEALTH%20RECORD>

^2^ Based on Population Estimates for Lower Layer Super Output Areas in England: <https://www.ons.gov.uk/peoplepopulationandcommunity/populationandmigration/populationestimates/datasets/lowersuperoutputareamidyearpopulationestimates>

**Table S4. Temporal Analysis of Hearing Loss Prevalence: Area Coverage and Estimated Population in Cheshire and Merseyside ICS Based on Time Series Clustering of Lower Super Output Areas (LSOAs), 2013-2022.**

| **Sub Integrated Care Board Locations^1^** | **Category**  **(rate of increase)** | **Area km^2^** | **Percentage** | **Estimated population of 50 years old and over residing in those areas^2^** | **Percentage of the population in the region** |
| --- | --- | --- | --- | --- | --- |
| Cheshire | High | 644.781017 | 30.90 | 99373 | 30.88% |
|  | Medium | 1124.160914 | 53.88 | 166315 | 51.69% |
|  | Low | 314.014049 | 15.05 | 56087 | 17.43% |
| Halton | High | 31.939092 | 40.38 | 27369 | 54.29% |
|  | Medium | 30.322383 | 38.34 | 21200 | 42.06% |
|  | Low | 16.833337 | 21.28 | 1840 | 3.65% |
| Knowsley | High | 1.591762 | 1.84 | 1164 | 2.00% |
|  | Medium | 33.10574 | 38.29 | 28147 | 48.44% |
|  | Low | 51.771909 | 59.87 | 28792 | 49.55% |
| Liverpool | High | 19.954883 | 17.84 | 28881 | 18.24% |
|  | Medium | 53.625772 | 47.95 | 74612 | 47.12% |
|  | Low | 38.263459 | 34.21 | 54863 | 34.65% |
| South Sefton | High | 16.12018 | 21.33 | 13133 | 19.21% |
|  | Medium | 50.224568 | 66.45 | 46127 | 67.47% |
|  | Low | 8.622667 | 11.41 | 9104 | 13.32% |
| Southport and Formby | High | 31.477044 | 38.86 | 21832 | 38.56% |
|  | Medium | 43.774975 | 54.05 | 27736 | 48.99% |
|  | Low | 3.989171 | 4.93 | 7043 | 12.44% |
| St Helens | High | 21.261166 | 15.59 | 21114 | 28.18% |
|  | Medium | 100.533794 | 73.72 | 43526 | 58.10% |
|  | Low | 14.586406 | 10.70 | 10277 | 13.72% |
| Warrington | High | 1.903943 | 1.05 | 1440 | 1.72% |
|  | Medium | 21.007035 | 11.63 | 10398 | 12.39% |
|  | Low | 157.712107 | 87.32 | 72054 | 85.89% |
| Wirral | High | 38.98512 | 24.23 | 26886 | 19.29% |
|  | Medium | 93.459108 | 58.08 | 86250 | 61.88% |
|  | Low | 24.470273 | 15.21 | 26254 | 18.83% |

^1^ Sub Integrated Care Board Location definition: <https://www.datadictionary.nhs.uk/supporting_information/sub_integrated_care_board_location.html#:~:text=A%20Sub%20Integrated%20Care%20Board%20Location%20is%20a,purposes%20and%20to%20support%20the%20ELECTRONIC%20HEALTH%20RECORD>

^2^ Based on Population Estimates for Lower Layer Super Output Areas in England: <https://www.ons.gov.uk/peoplepopulationandcommunity/populationandmigration/populationestimates/datasets/lowersuperoutputareamidyearpopulationestimates>

**Table S5. Geographically Weighted Regression Analysis: Exploring the Relationship between Index of Multiple Deprivation (IMD) 2019 and Prevalence of Hearing Loss in 2020.**

| **Sub Integrated Care Board Locations** | **Mean Local R-squared** | **Median Local R-squared** | **Min Local R-squared** | **Max Local R-squared** | **Range Local R-squared** | **Standard Deviation**  **Local R-squared** |
| --- | --- | --- | --- | --- | --- | --- |
| Cheshire | 0.13 | 0.11 | -0.23 | 0.35 | 0.58 | 0.09 |
| Halton | 0.21 | 0.21 | 0.18 | 0.25 | 0.07 | 0.01 |
| Knowsley | 0.18 | 0.18 | 0.16 | 0.22 | 0.05 | 0.01 |
| Liverpool | 0.20 | 0.21 | 0.17 | 0.23 | 0.07 | 0.02 |
| South Sefton | 0.24 | 0.24 | 0.20 | 0.29 | 0.09 | 0.02 |
| Southport and Formby | 0.10 | 0.06 | 0.04 | 0.29 | 0.25 | 0.08 |
| St Helens | 0.15 | 0.15 | 0.11 | 0.18 | 0.07 | 0.02 |
| Warrington | 0.15 | 0.17 | -0.12 | 0.27 | 0.39 | 0.08 |
| Wirral | 0.27 | 0.27 | 0.24 | 0.29 | 0.05 | 0.01 |

**Table S6. Summary Statistics of Median Age in Lower Super Output Areas (LSOAs): Cheshire and Merseyside ICS Sub Integrated Care Board Locations, 2020.**

| **Sub Integrated Care Board Locations** | **Mean** | **Median** | **Minimum** | **Maximum** | **Range** | **Standard Deviation** |
| --- | --- | --- | --- | --- | --- | --- |
| Cheshire | 64.69 | 65 | 54 | 71 | 17 | 2.75 |
| Halton | 63.91 | 64 | 58 | 69 | 11 | 2.30 |
| Knowsley | 63.17 | 63 | 60 | 68 | 8 | 1.84 |
| Liverpool | 63.17 | 63 | 57 | 70 | 13 | 2.10 |
| South Sefton | 63.94 | 64 | 60 | 71 | 11 | 2.28 |
| Southport and Formby | 66.25 | 66.5 | 61 | 73 | 12 | 3.28 |
| St Helens | 64.53 | 64 | 59 | 72 | 13 | 2.68 |
| Warrington | 63.69 | 63 | 57 | 72 | 15 | 3.07 |
| Wirral | 64.70 | 65 | 59 | 71 | 12 | 2.64 |

**Table S7. Geographically Weighted Regression Analysis: Investigating the Relationship between Median Age of Lower Super Output Areas (LSOAs) in Cheshire and Merseyside ICS Sub Integrated Care Board Locations and Prevalence of Hearing Loss in 2020.**

| **Sub Integrated Care Board Locations** | **Mean** | **Median** | **Minimum** | **Maximum** | **Range** | **Standard Deviation** |
| --- | --- | --- | --- | --- | --- | --- |
| Cheshire | 0.42 | 0.41 | 0.16 | 0.75 | 0.59 | 0.08 |
| Halton | 0.40 | 0.40 | 0.38 | 0.45 | 0.07 | 0.01 |
| Knowsley | 0.38 | 0.38 | 0.37 | 0.41 | 0.05 | 0.01 |
| Liverpool | 0.41 | 0.41 | 0.37 | 0.45 | 0.08 | 0.02 |
| South Sefton | 0.43 | 0.43 | 0.40 | 0.48 | 0.08 | 0.01 |
| Southport and Formby | 0.55 | 0.56 | 0.48 | 0.58 | 0.10 | 0.03 |
| St Helens | 0.37 | 0.37 | 0.36 | 0.38 | 0.03 | 0.00 |
| Warrington | 0.36 | 0.39 | 0.12 | 0.46 | 0.34 | 0.07 |
| Wirral | 0.49 | 0.49 | 0.46 | 0.53 | 0.07 | 0.02 |

**Table S8. Summary Statistics of Hearing Loss Prevalence in Sub Integrated Care Board Locations within Cheshire and Merseyside ICS: 2013-2027**

| **Sub Integrated Care Board Locations** | **Years** | **Minimum** | **Maximum** | **Mean** | **Standard Deviation** | **Median** | **Range** | **Variance** |
| --- | --- | --- | --- | --- | --- | --- | --- | --- |
| Cheshire | Decade average 2013-2022 | 1.80 | 11.44 | 6.13 | 1.55 | 6.10 | 9.63 | 2.50 |
|  | 2013 | 0.87 | 8.25 | 3.87 | 1.10 | 3.82 | 7.38 | 1.21 |
|  | 2014 | 0.99 | 8.16 | 4.37 | 1.20 | 4.32 | 7.17 | 1.44 |
|  | 2015 | 1.11 | 9.20 | 4.87 | 1.31 | 4.83 | 8.09 | 1.70 |
|  | 2016 | 1.23 | 10.27 | 5.35 | 1.40 | 5.30 | 9.04 | 1.97 |
|  | 2017 | 1.73 | 11.44 | 5.85 | 1.51 | 5.84 | 9.71 | 2.27 |
|  | 2018 | 1.97 | 11.40 | 6.37 | 1.58 | 6.39 | 9.43 | 2.50 |
|  | 2019 | 2.27 | 12.25 | 6.90 | 1.66 | 6.93 | 9.98 | 2.75 |
|  | 2020 | 2.44 | 13.32 | 7.50 | 1.80 | 7.48 | 10.88 | 3.25 |
|  | 2021 | 2.69 | 14.46 | 7.88 | 1.92 | 7.83 | 11.77 | 3.70 |
|  | 2022 | 2.71 | 15.61 | 8.33 | 2.06 | 8.25 | 12.90 | 4.25 |
|  | Predicted 2023 | 2.89 | 17.14 | 8.75 | 2.24 | 8.68 | 14.24 | 5.03 |
|  | Predicted 2024 | 3.01 | 19.05 | 9.12 | 2.42 | 8.99 | 16.04 | 5.83 |
|  | Predicted 2025 | 3.11 | 21.14 | 9.46 | 2.61 | 9.25 | 18.04 | 6.80 |
|  | Predicted 2026 | 3.19 | 23.42 | 9.79 | 2.82 | 9.50 | 20.23 | 7.96 |
|  | Predicted 2027 | 3.27 | 25.89 | 10.09 | 3.06 | 9.68 | 22.62 | 9.36 |
| Halton | Decade average 2013-2022 | 2.59 | 10.06 | 6.39 | 1.56 | 6.38 | 7.47 | 2.55 |
|  | 2013 | 1.70 | 6.55 | 3.74 | 1.05 | 3.60 | 4.85 | 1.11 |
|  | 2014 | 1.81 | 7.48 | 4.26 | 1.17 | 4.15 | 5.67 | 1.36 |
|  | 2015 | 2.06 | 7.82 | 4.87 | 1.26 | 4.79 | 5.76 | 1.60 |
|  | 2016 | 2.20 | 8.62 | 5.51 | 1.40 | 5.48 | 6.42 | 1.97 |
|  | 2017 | 2.35 | 9.12 | 6.07 | 1.48 | 6.03 | 6.77 | 2.19 |
|  | 2018 | 2.49 | 9.94 | 6.71 | 1.58 | 6.86 | 7.45 | 2.49 |
|  | 2019 | 2.98 | 10.97 | 7.33 | 1.68 | 7.39 | 7.99 | 2.82 |
|  | 2020 | 3.25 | 12.26 | 8.01 | 1.87 | 8.05 | 9.01 | 3.50 |
|  | 2021 | 3.42 | 13.31 | 8.45 | 1.99 | 8.52 | 9.89 | 3.96 |
|  | 2022 | 3.61 | 14.49 | 8.96 | 2.12 | 8.93 | 10.88 | 4.50 |
|  | Predicted 2023 | 3.86 | 15.76 | 9.37 | 2.28 | 9.35 | 11.90 | 5.20 |
|  | Predicted 2024 | 4.04 | 17.11 | 9.78 | 2.45 | 9.84 | 13.07 | 6.01 |
|  | Predicted 2025 | 4.20 | 18.54 | 10.15 | 2.64 | 10.05 | 14.34 | 6.96 |
|  | Predicted 2026 | 4.34 | 20.05 | 10.50 | 2.84 | 10.38 | 15.71 | 8.06 |
|  | Predicted 2027 | 4.46 | 21.65 | 10.81 | 3.05 | 10.56 | 17.19 | 9.33 |
| Knowsley | Decade average 2013-2022 | 1.48 | 7.56 | 4.29 | 1.07 | 4.30 | 6.08 | 1.19 |
|  | 2013 | 0.81 | 4.79 | 2.55 | 0.68 | 2.60 | 3.98 | 0.46 |
|  | 2014 | 0.85 | 5.51 | 2.91 | 0.78 | 2.93 | 4.66 | 0.62 |
|  | 2015 | 0.95 | 6.45 | 3.31 | 0.90 | 3.39 | 5.50 | 0.80 |
|  | 2016 | 1.05 | 6.80 | 3.72 | 0.99 | 3.79 | 5.75 | 0.97 |
|  | 2017 | 1.08 | 7.51 | 4.11 | 1.09 | 4.19 | 6.43 | 1.19 |
|  | 2018 | 1.33 | 7.73 | 4.51 | 1.14 | 4.53 | 6.40 | 1.30 |
|  | 2019 | 1.79 | 8.24 | 4.92 | 1.20 | 4.94 | 6.45 | 1.43 |
|  | 2020 | 2.02 | 8.78 | 5.31 | 1.25 | 5.29 | 6.76 | 1.57 |
|  | 2021 | 2.37 | 9.48 | 5.62 | 1.32 | 5.50 | 7.11 | 1.74 |
|  | 2022 | 2.52 | 10.31 | 5.96 | 1.37 | 5.86 | 7.79 | 1.87 |
|  | Predicted 2023 | 2.72 | 10.61 | 6.24 | 1.41 | 6.10 | 7.89 | 1.98 |
|  | Predicted 2024 | 2.77 | 11.14 | 6.51 | 1.47 | 6.41 | 8.37 | 2.16 |
|  | Predicted 2025 | 2.80 | 11.67 | 6.76 | 1.55 | 6.75 | 8.87 | 2.41 |
|  | Predicted 2026 | 2.81 | 12.19 | 7.00 | 1.66 | 7.05 | 9.38 | 2.74 |
|  | Predicted 2027 | 2.82 | 12.70 | 7.22 | 1.79 | 7.20 | 9.88 | 3.21 |
| Liverpool | Decade average 2013-2022 | 0.84 | 8.21 | 4.43 | 1.56 | 4.51 | 7.36 | 2.59 |
|  | 2013 | 0.49 | 5.19 | 2.55 | 0.93 | 2.56 | 4.70 | 0.86 |
|  | 2014 | 0.65 | 5.77 | 2.97 | 1.07 | 3.02 | 5.12 | 1.15 |
|  | 2015 | 0.65 | 6.53 | 3.40 | 1.21 | 3.49 | 5.88 | 1.48 |
|  | 2016 | 0.72 | 7.24 | 3.83 | 1.36 | 3.92 | 6.52 | 1.85 |
|  | 2017 | 0.78 | 7.89 | 4.26 | 1.52 | 4.35 | 7.11 | 2.31 |
|  | 2018 | 0.84 | 8.44 | 4.68 | 1.66 | 4.77 | 7.60 | 2.75 |
|  | 2019 | 0.97 | 9.22 | 5.09 | 1.79 | 5.16 | 8.25 | 3.22 |
|  | 2020 | 1.07 | 9.79 | 5.52 | 1.92 | 5.60 | 8.72 | 3.68 |
|  | 2021 | 1.12 | 10.54 | 5.85 | 2.02 | 5.97 | 9.42 | 4.08 |
|  | 2022 | 1.16 | 11.47 | 6.20 | 2.13 | 6.26 | 10.31 | 4.53 |
|  | Predicted 2023 | 1.15 | 11.98 | 6.49 | 2.20 | 6.57 | 10.84 | 4.86 |
|  | Predicted 2024 | 1.16 | 12.68 | 6.77 | 2.29 | 6.77 | 11.52 | 5.24 |
|  | Predicted 2025 | 1.16 | 13.37 | 7.03 | 2.37 | 7.03 | 12.21 | 5.62 |
|  | Predicted 2026 | 1.17 | 14.06 | 7.27 | 2.45 | 7.26 | 12.89 | 6.02 |
|  | Predicted 2027 | 1.18 | 14.74 | 7.50 | 2.54 | 7.52 | 13.56 | 6.45 |
| South Sefton | Decade average 2013-2022 | 2.98 | 8.81 | 5.51 | 1.21 | 5.40 | 5.84 | 1.53 |
|  | 2013 | 1.54 | 5.63 | 3.29 | 0.83 | 3.21 | 4.09 | 0.69 |
|  | 2014 | 1.63 | 6.37 | 3.76 | 0.91 | 3.68 | 4.74 | 0.83 |
|  | 2015 | 2.27 | 6.99 | 4.29 | 0.93 | 4.25 | 4.72 | 0.87 |
|  | 2016 | 2.58 | 7.74 | 4.81 | 1.06 | 4.68 | 5.16 | 1.13 |
|  | 2017 | 3.06 | 8.58 | 5.34 | 1.17 | 5.30 | 5.52 | 1.37 |
|  | 2018 | 3.37 | 9.52 | 5.85 | 1.28 | 5.79 | 6.15 | 1.63 |
|  | 2019 | 3.57 | 9.72 | 6.32 | 1.34 | 6.20 | 6.15 | 1.79 |
|  | 2020 | 3.87 | 10.42 | 6.76 | 1.41 | 6.62 | 6.55 | 2.00 |
|  | 2021 | 3.87 | 11.22 | 7.14 | 1.51 | 6.99 | 7.35 | 2.29 |
|  | 2022 | 4.00 | 11.94 | 7.58 | 1.63 | 7.27 | 7.94 | 2.67 |
|  | Predicted 2023 | 4.01 | 12.84 | 7.83 | 1.69 | 7.53 | 8.83 | 2.87 |
|  | Predicted 2024 | 4.03 | 13.72 | 8.12 | 1.79 | 7.74 | 9.70 | 3.21 |
|  | Predicted 2025 | 4.04 | 14.63 | 8.40 | 1.90 | 8.03 | 10.59 | 3.61 |
|  | Predicted 2026 | 4.05 | 15.56 | 8.65 | 2.02 | 8.33 | 11.52 | 4.08 |
|  | Predicted 2027 | 4.05 | 16.52 | 8.88 | 2.15 | 8.54 | 12.47 | 4.64 |
| Southport and Formby | Decade average 2013-2022 | 3.89 | 11.08 | 6.21 | 1.53 | 6.08 | 7.19 | 2.51 |
|  | 2013 | 2.18 | 6.68 | 3.86 | 0.92 | 3.81 | 4.50 | 0.85 |
|  | 2014 | 2.51 | 7.55 | 4.34 | 1.03 | 4.27 | 5.04 | 1.07 |
|  | 2015 | 2.76 | 8.38 | 4.89 | 1.18 | 4.87 | 5.62 | 1.38 |
|  | 2016 | 3.44 | 9.42 | 5.44 | 1.33 | 5.40 | 5.98 | 1.77 |
|  | 2017 | 3.71 | 10.18 | 5.92 | 1.44 | 5.84 | 6.47 | 2.07 |
|  | 2018 | 4.15 | 11.24 | 6.45 | 1.56 | 6.47 | 7.09 | 2.43 |
|  | 2019 | 4.51 | 12.53 | 6.97 | 1.70 | 6.75 | 8.02 | 2.90 |
|  | 2020 | 4.99 | 13.27 | 7.58 | 1.82 | 7.22 | 8.28 | 3.30 |
|  | 2021 | 5.21 | 15.15 | 8.05 | 2.06 | 7.82 | 9.94 | 4.24 |
|  | 2022 | 5.44 | 16.36 | 8.62 | 2.26 | 8.33 | 10.92 | 5.09 |
|  | Predicted 2023 | 5.61 | 17.88 | 9.02 | 2.34 | 8.82 | 12.27 | 5.48 |
|  | Predicted 2024 | 5.73 | 19.54 | 9.49 | 2.57 | 9.29 | 13.81 | 6.62 |
|  | Predicted 2025 | 5.81 | 21.33 | 9.94 | 2.83 | 9.75 | 15.52 | 8.00 |
|  | Predicted 2026 | 5.88 | 23.27 | 10.38 | 3.11 | 10.11 | 17.39 | 9.68 |
|  | Predicted 2027 | 5.92 | 25.35 | 10.82 | 3.42 | 10.49 | 19.43 | 11.70 |
| St Helens | Decade average 2013-2022 | 2.62 | 9.49 | 5.37 | 1.26 | 5.23 | 6.87 | 1.68 |
|  | 2013 | 1.12 | 6.10 | 2.93 | 0.76 | 2.90 | 4.98 | 0.57 |
|  | 2014 | 1.82 | 6.81 | 3.40 | 0.81 | 3.29 | 4.99 | 0.66 |
|  | 2015 | 2.19 | 7.48 | 4.09 | 0.99 | 3.98 | 5.29 | 0.98 |
|  | 2016 | 2.48 | 8.19 | 4.73 | 1.17 | 4.53 | 5.71 | 1.36 |
|  | 2017 | 2.66 | 9.25 | 5.27 | 1.25 | 5.06 | 6.59 | 1.57 |
|  | 2018 | 2.83 | 10.06 | 5.75 | 1.32 | 5.61 | 7.23 | 1.75 |
|  | 2019 | 3.03 | 10.74 | 6.22 | 1.42 | 6.12 | 7.71 | 2.02 |
|  | 2020 | 3.34 | 11.33 | 6.74 | 1.54 | 6.61 | 7.99 | 2.36 |
|  | 2021 | 3.35 | 12.23 | 7.09 | 1.62 | 6.94 | 8.88 | 2.64 |
|  | 2022 | 3.37 | 12.73 | 7.46 | 1.70 | 7.28 | 9.36 | 2.90 |
|  | Predicted 2023 | 3.38 | 12.87 | 7.73 | 1.75 | 7.58 | 9.48 | 3.06 |
|  | Predicted 2024 | 3.39 | 13.09 | 7.98 | 1.82 | 7.99 | 9.70 | 3.31 |
|  | Predicted 2025 | 3.40 | 13.26 | 8.21 | 1.89 | 8.18 | 9.86 | 3.57 |
|  | Predicted 2026 | 3.40 | 13.38 | 8.41 | 1.97 | 8.33 | 9.98 | 3.86 |
|  | Predicted 2027 | 3.40 | 13.72 | 8.59 | 2.05 | 8.40 | 10.32 | 4.19 |
| Warrington | Decade average 2013-2022 | 1.73 | 9.01 | 4.02 | 1.22 | 3.88 | 7.29 | 1.56 |
|  | 2013 | 1.18 | 4.79 | 2.87 | 0.80 | 2.82 | 3.61 | 0.65 |
|  | 2014 | 1.26 | 5.30 | 3.12 | 0.87 | 3.00 | 4.04 | 0.76 |
|  | 2015 | 1.48 | 6.64 | 3.37 | 0.96 | 3.28 | 5.16 | 0.92 |
|  | 2016 | 1.53 | 7.89 | 3.65 | 1.07 | 3.56 | 6.36 | 1.13 |
|  | 2017 | 1.70 | 8.36 | 3.88 | 1.15 | 3.73 | 6.66 | 1.33 |
|  | 2018 | 1.83 | 9.74 | 4.11 | 1.24 | 3.89 | 7.91 | 1.55 |
|  | 2019 | 1.87 | 10.82 | 4.38 | 1.36 | 4.18 | 8.95 | 1.86 |
|  | 2020 | 1.99 | 11.51 | 4.70 | 1.45 | 4.53 | 9.52 | 2.11 |
|  | 2021 | 2.19 | 12.24 | 4.95 | 1.56 | 4.75 | 10.05 | 2.42 |
|  | 2022 | 2.24 | 12.85 | 5.21 | 1.68 | 5.04 | 10.61 | 2.82 |
|  | Predicted 2023 | 2.38 | 13.49 | 5.47 | 1.88 | 5.25 | 11.11 | 3.53 |
|  | Predicted 2024 | 2.50 | 14.00 | 5.72 | 2.00 | 5.36 | 11.49 | 3.99 |
|  | Predicted 2025 | 2.61 | 14.43 | 5.97 | 2.12 | 5.53 | 11.81 | 4.50 |
|  | Predicted 2026 | 2.62 | 14.77 | 6.22 | 2.26 | 5.76 | 12.15 | 5.09 |
|  | Predicted 2027 | 2.63 | 15.04 | 6.47 | 2.41 | 6.04 | 12.41 | 5.79 |
| Wirral | Decade average 2013-2022 | 2.87 | 13.23 | 5.88 | 1.75 | 5.58 | 10.36 | 3.10 |
|  | 2013 | 1.73 | 10.03 | 3.71 | 1.44 | 3.38 | 8.30 | 2.08 |
|  | 2014 | 2.05 | 10.74 | 4.21 | 1.52 | 3.88 | 8.69 | 2.32 |
|  | 2015 | 2.15 | 11.24 | 4.69 | 1.58 | 4.40 | 9.09 | 2.48 |
|  | 2016 | 2.51 | 12.33 | 5.20 | 1.68 | 4.90 | 9.82 | 2.82 |
|  | 2017 | 2.86 | 13.30 | 5.70 | 1.73 | 5.37 | 10.44 | 2.99 |
|  | 2018 | 3.18 | 13.75 | 6.17 | 1.78 | 5.84 | 10.57 | 3.16 |
|  | 2019 | 3.37 | 14.53 | 6.64 | 1.84 | 6.43 | 11.16 | 3.40 |
|  | 2020 | 3.48 | 15.15 | 7.13 | 1.91 | 6.91 | 11.67 | 3.65 |
|  | 2021 | 3.51 | 15.47 | 7.46 | 1.97 | 7.20 | 11.96 | 3.89 |
|  | 2022 | 3.87 | 15.77 | 7.87 | 2.05 | 7.53 | 11.90 | 4.19 |
|  | Predicted 2023 | 3.82 | 16.01 | 8.19 | 2.13 | 7.88 | 12.19 | 4.53 |
|  | Predicted 2024 | 3.87 | 16.18 | 8.49 | 2.22 | 8.28 | 12.31 | 4.94 |
|  | Predicted 2025 | 3.91 | 16.32 | 8.77 | 2.33 | 8.55 | 12.41 | 5.44 |
|  | Predicted 2026 | 3.94 | 16.49 | 9.04 | 2.46 | 8.77 | 12.55 | 6.03 |
|  | Predicted 2027 | 3.96 | 17.51 | 9.28 | 2.60 | 8.96 | 13.56 | 6.74 |

**
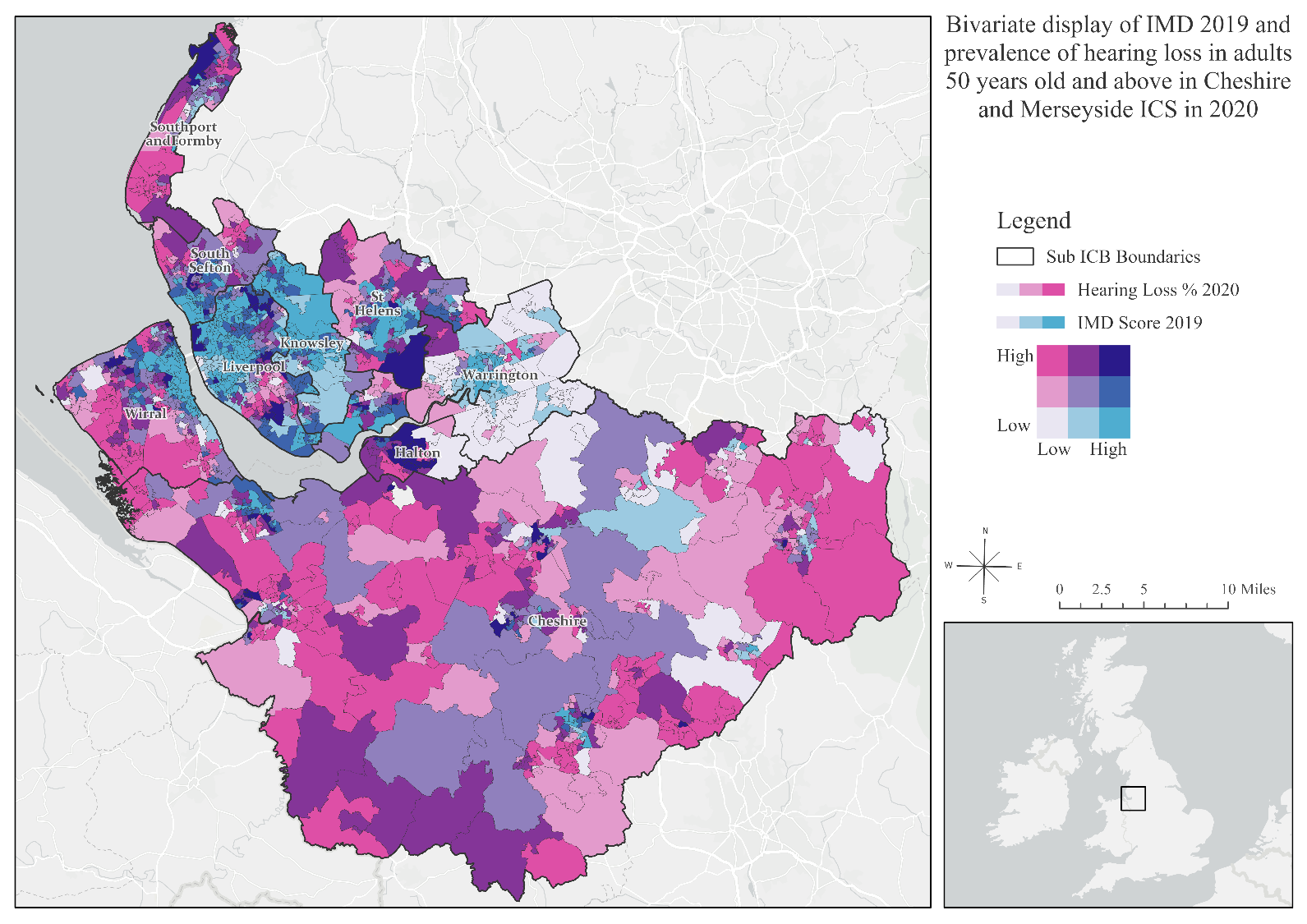
Fig. S1. Bivariate Analysis: Mapping IMD 2019 and Prevalence of Hearing Loss in Adults (50+) in Cheshire and Merseyside ICS, 2020**


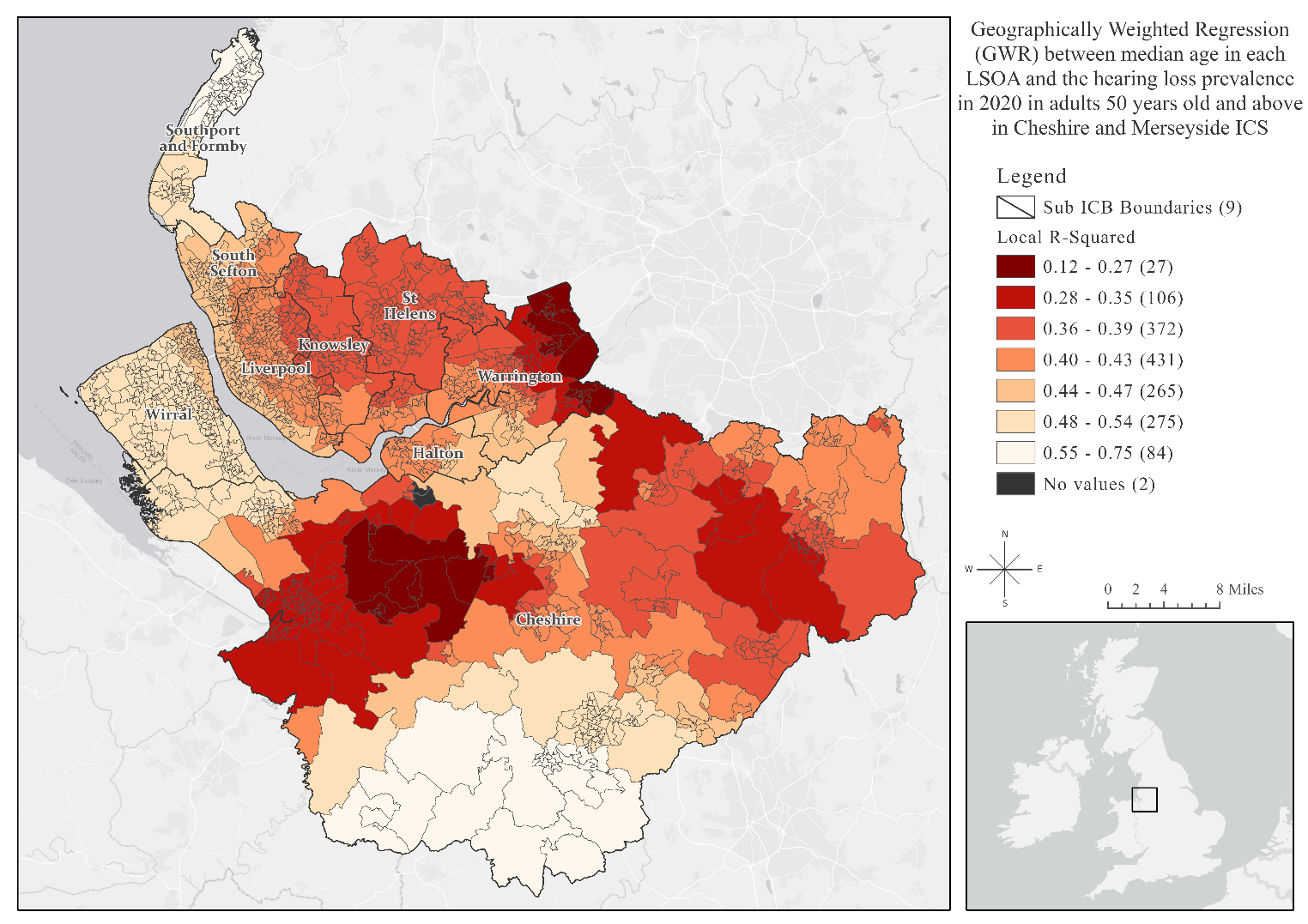


**Fig. S2. Geographically Weighted Regression Analysis: Exploring the Relationship between Median Age in LSOAs and Hearing Loss Prevalence in Adults (50+) in Cheshire and Merseyside ICS, 2020**
